# Supplementary material for: Blood Plasma Exosomes Contain Circulating DNA in Their Crown
Source: Diagnostics (Basel). 2022 Mar 30;12(4):854. doi: 10.3390/diagnostics12040854 (PMC9027845; doi:10.3390/diagnostics12040854)
Supplement: Supplementary file 1 [file diagnostics-12-00854-s001.zip › Table S1.pdf]

Table S1. Exosomal proteins identified in the HF plasma*

Uniprot ID	Protein	Gene
P0DOX6	Immunoglobulin mu heavy chain	
P01023	*Alpha-2-macroglobulin*	*A2M*
Q4LEZ3	*Alanine and arginine-rich domain-containing protein*	*AARD*
O14672	***Disintegrin and metalloproteinase domain-containing protein 10***	***ADAM10***
P78536	***Disintegrin and metalloproteinase domain-containing protein 17***	***ADAM17***
P02765	***Alpha-2-HS-glycoprotein***	***AHSG***
O95831	*Apoptosis-inducing factor 1, mitochondrial*	*AIFM1*
O14862	Interferon-inducible protein AIM2	AIM2
P02768	***Serum albumin***	***ALB***
P02760	***Alpha-1-microglycoprotein***	***AMBP***
P23109	AMP deaminase 1	AMPD1
P02647	***Apolipoprotein A-I***	***APOA1***
P06727	Apolipoprotein A-IV	APOA4
P04114	***Apolipoprotein B-100***	***APOB***
P02649	*Apolipoprotein E*	*APOE*
O75531	***Barrier-to-autointegration factor***	***BANF1***
Q99728	*BRCA1-associated RING domain protein 1*	*BARD1*
P01024	***Complement C3***	***C3***
Q8N5S9	Calcium/calmodulin-dependent protein kinase kinase 1	CAMKK1
Q96PX6	*Coiled-coil domain-containing protein 85A*	*CCDC85A*
P25063	***Signal transducer CD24***	***CD24***
P08962	***CD63 antigen***	***CD63***
P60033	***CD81 antigen***	***CD81***
P21926	***CD9 antigen***	***CD9***
A0A1B0GU71	Uncharacterized protein CFAP97D2	CFAP97D
P08603	*Complement factor H*	*CFH*
Q8TDI0	*Chromodomain-helicase-DNA-binding protein 5*	*CHD5*
F8VUA8	Uncharacterized protein CLBA1	CLBA1
P10909	***Clusterin***	***CLU***
P61201	*COP9 signalosome complex subunit 2*	*COPS2*
Q6QEF8	*Coronin-6*	*CORO6*
P12074	Cytochrome c oxidase subunit 6A1, mitochondrial	COX6A1
Q8TES7	***Fas-binding factor 1***	***FBF1***
P02671	*Fibrinogen alpha chain*	*FGA*
P02675	*Fibrinogen beta chain*	*FGB*
O15520	*Fibroblast growth factor 10*	*FGF10*
P02679	***Fibrinogen gamma chain***	***FGG***
P02751	*Fibronectin*	*FN1*
Q96DB9	*FXYD domain-containing ion transport regulator 5*	*FXYD5*
O60861	*Growth arrest-specific protein 7*	*GAS7*
P06396	***Gelsolin***	***GSN***
O96004	Heart- and neural crest derivatives-expressed protein 1	HAND1
P68871	***Hemoglobin subunit beta***	***HBB***
P00738	***Haptoglobin***	***HP***
P00739	***Haptoglobin-related protein***	***HPR***
P02790	***Hemopexin***	***HPX***
P01859	Ig gamma-2 chain C region	IGHG2
P01871	Ig mu chain C region	IGHM

P01834	Ig kappa chain C region	IGKC
A0M8Q6	Ig lambda-7 chain C region	IGLC7
P02750	***Leucine-rich alpha-2-glycoprotein***	***LRG***
H0YGS3	Microfibrillar-associated protein 5	MFAP5
Q16674	*Melanoma-derived growth regulatory protein*	*MIA*
Q8N159	*N-acetylglutamate synthase, mitochondrial*	*NAGS*
P60323	Nanos homolog 3	NANOS3
P55209	*Nucleosome assembly protein 1-like 1*	*NAP1L1*
O43678	*NADH dehydrogenase [ubiquinone] 1 alpha subcomplex subunit 2*	*NDUFA2*
Q9GZT8	*NIF3-like protein 1*	*NIF3L1*
Q9HCQ7	*Pro-FMRFamide-related neuropeptide VF*	*NPVF*
P56373	P2X purinoceptor 3	P2RX3
O00329	*Phosphatidylinositol 4,5-bisphosphate 3-kinase catalytic subunit delta isoform*	*PIK3CD*
P78356	*Phosphatidylinositol 4-kinase type 2-beta*	*PIP4K2B*
Q6IQ23	*Pleckstrin homology domain-containing family A member 7*	*PLEKHA7*
P11801	*Serine/threonine-protein kinase H1*	*PSKH1*
Q9UL26	*Ras-related protein Rab-22A*	*RAB22A*
Q8WXH6	Ras-related protein Rab-40A	RAB40A
P61224	*Ras-related GTP-binding protein B*	*RAP1B*
Q8TDF6	RAS guanyl-releasing protein 4	RASGRP4
P50749	*Ras association domain-containing protein 2*	*RASSF2*
O75526	*RNA-binding motif protein, X-linked-like-2*	*RBMXL2*
P40938	*Replication factor C subunit 3*	*RFC3*
Q969K3	*E3 ubiquitin-protein ligase RNF34*	*RNF34*
Q13424	Alpha-1-syntrophin	SNTA1
P02787	Serotransferrin	TF
Q9NUR3	Transmembrane protein 74B	TMEM74B
O43557	Tumor necrosis factor ligand superfamily member 14	TNFSF14
O43399	***Tumor protein D54***	***TPD52L2***
P02766	***Transthyretin***	***TTR***
P43403	*Tyrosine-protein kinase ZAP-70*	*ZAP70*
Q15776	Zinc finger protein with KRAB and SCAN domains 8	ZKSCAN8
Q9Y2P0	Zinc finger protein 835	ZNF835
O43309	Zinc finger and SCAN domain-containing protein 12	ZSCAN12

* - universal plasma exosome proteins of HFs and BCPs are in bold type, proteins previously identified in the Vesiclepedia database are marked in italics.
