# Supplementary material for: Blood Plasma Exosomes Contain Circulating DNA in Their Crown
Source: Diagnostics (Basel). 2022 Mar 30;12(4):854. doi: 10.3390/diagnostics12040854 (PMC9027845; doi:10.3390/diagnostics12040854)
Supplement: Supplementary file 1 [file diagnostics-12-00854-s001.zip › Table S2.pdf]

Table S2. Exosomal proteins identified in the BCP plasma*

Uniprot	Protein	Gene Name
P69905	Haptoglobin alpha chain	
O75027	*ATP-binding cassette sub-family B member 7, mitochondrial*	*ABCB7*
P11310	*Medium-chain specific acyl-CoA dehydrogenase, mitochondrial*	*ACADM*
Q53FZ2	Acyl-coenzyme A synthetase ACSM3, mitochondrial	ACSM3
O14672	***Disintegrin and metalloproteinase domain-containing protein 10***	***ADAM10***
P78536	***Disintegrin and metalloproteinase domain-containing protein 17***	***ADAM17***
P02765	***Alpha-2-HS-glycoprotein***	***AHSG***
P02768	***Serum albumin***	***ALB***
P02760	***Alpha-1-microglycoprotein***	***AMBP***
Q5CZ79	Ankyrin repeat domain-containing protein 20B	ANKRD20A8P
Q9BYT9	*Anoctamin-3*	*ANO3*
P02647	***Apolipoprotein A-I***	***APOA1***
P06727	Apolipoprotein A-IV	APOA4
P04114	***Apolipoprotein B-100***	***APOB***
Q9P291	*Armadillo repeat-containing X-linked protein 1*	*ARMCX1*
O75531	***Barrier-to-autointegration factor***	***BANF1***
P18075	*Bone morphogenetic protein 7*	*BMP7*
P01024	***Complement C3***	***C3***
O00555	*Voltage-dependent P/Q-type calcium channel subunit alpha-1A*	*CACNA1A*
P17655	*Calpain-2 catalytic subunit*	*CAPN2*
Q5M9N0	*Coiled-coil domain-containing protein 158*	*CCDC158*
P25063	***Signal transducer CD24***	***CD24***
P08962	***CD63 antigen***	***CD63***
P60033	***CD81 antigen***	***CD81***
P21926	***CD9 antigen***	***CD9***
Q5VT06	*Centrosome-associated protein 350*	*CEP350*
P17540	*Creatine kinase S-type, mitochondrial*	*CKMT2*
P10909	***Clusterin***	***CLU***
P02489	*Alpha-crystallin A chain*	*CRYAA*
A0A140G945	Alpha-crystallin A2 chain	CRYAA2
P02511	*Alpha-crystallin B chain*	*CRYAB*
O00429	*Dynamin-1-like protein*	*DNM1L*
Q9Y4J8	*Dystrobrevin alpha*	*DTNA*
Q8N9H8	*Exonuclease mut-7 homolog*	*EXD3*
Q8IYI6	*Exocyst complex component 8*	*EXOC8*
Q8TES7	***Fas-binding factor 1***	***FBF1***
P02679	***Fibrinogen gamma chain***	***FGG***
P06396	***Gelsolin***	***GSN***
Q9NYZ3	*G2 and S phase-expressed protein 1*	*GTSE1*
P68871	***Hemoglobin subunit beta***	***HBB***
Q9NWT6	*Hypoxia-inducible factor 1-alpha inhibitor*	*HIF1AN*
P00738	***Haptoglobin***	***HP***
P00739	***Haptoglobin-related protein***	***HPR***
P02790	***Hemopexin***	***HPX***

P11717	*Cation-independent mannose-6-phosphate receptor*	*IGF2R*
P01876	Immunoglobulin heavy constant alpha 1	IGHA1
P01857	Immunoglobulin heavy constant gamma 1	IGHG1
P01834	Immunoglobulin kappa constant	IGKC
P01619	Immunoglobulin kappa light chain	IGKV3-20
Q9Y2W7	*Calsenilin*	*KCNIP3*
Q96Q89	*Kinesin-like protein KIF20B*	*KIF20B*
O15066	*Kinesin-like protein KIF3B*	*KIF3B*
Q9BVG8	*Kinesin-like protein KIFC3*	*KIFC3*
P02750	***Leucine-rich alpha-2-glycoprotein***	***LRG***
Q96EY8	*Corrinoid adenosyltransferase*	*MMAB*
Q86TS9	*39S ribosomal protein L52, mitochondrial*	*MRPL52*
P11586	*C-1-tetrahydrofolate synthase, cytoplasmic*	*MTHFD1*
Q8NFA2	NADPH oxidase organizer 1	NOXO1
P0CE72	Oncomodulin-1	OCM
Q29RF7	*Sister chromatid cohesion protein PDS5 homolog A*	*PDS5A*
Q8WXW3	*Progesterone-induced-blocking factor 1*	*PIBF1*
O15514	DNA-directed RNA polymerase II subunit RPB4	POLR2D
Q8NEY8	Periphilin-1	PPHLN1
Q969Q5	*Ras-related protein Rab-24*	*RAB24*
P46779	*60S ribosomal protein L28*	*RPL28*
P01009	*Alpha-1-antitrypsin*	*SERPINA1*
Q5VZ18	SH2 domain-containing adapter protein E	SHE
Q9BT92	*Trichoplein keratin filament-binding protein*	*TCHP*
P02787	*Serotransferrin*	*TF*
Q9NQ34	*Transmembrane protein 9B*	*TMEM9B*
O43399	***Tumor protein D54***	***TPD52L2***
O15050	*TPR and ankyrin repeat-containing protein 1*	*TRANK1*
P02766	***Transthyretin***	***TTR***
Q9UKW4	*Guanine nucleotide exchange factor VAV3*	*VAV3*
Q96RL7	*Vacuolar protein sorting-associated protein 13A*	*VPS13A*
Q969S3	*Zinc finger protein 622*	*ZNF622*
Q14966	*Zinc finger protein 638*	*ZNF638*

* - universal plasma exosome proteins of HFs and BCPs are in bold type, proteins previously identified in the Vesiclepedia database are marked in italics.
